# Panitumumab Use in Metastatic Colorectal Cancer and Patterns of *KRAS* Testing: Results from a Europe-Wide Physician Survey and Medical Records Review

**DOI:** 10.1371/journal.pone.0140717

**Published:** 2015-10-22

**Authors:** Jörg Trojan, Laurent Mineur, Jiří Tomášek, Etienne Rouleau, Pavel Fabian, Giovanna de Maglio, Pilar García-Alfonso, Giuseppe Aprile, Aliki Taylor, George Kafatos, Gerald Downey, Jan-Henrik Terwey, J. Han van Krieken

**Affiliations:** 1 University Hospital, Frankfurt, Germany; 2 Institute Sainte Catherine, Avignon, France; 3 Masaryk Memorial Cancer Institute, Brno, Czech Republic; 4 Curie Institute, Paris, France; 5 University and General Hospital, Udine, Italy; 6 Gregorio Marañón Hospital, Madrid, Spain; 7 Amgen Ltd, Uxbridge, UK; 8 Amgen (Europe) GmbH, Zug Switzerland; 9 Radboud University Medical Center, Nijmegen, Netherlands; University of Naples Federico II, Naples, Italy, ITALY

## Abstract

**Background:**

From 2008–2013, the European indication for panitumumab required that patients’ tumor *KRAS* exon 2 mutation status was known prior to starting treatment. To evaluate physician awareness of panitumumab prescribing information and how physicians prescribe panitumumab in patients with metastatic colorectal cancer (mCRC), two European multi-country, cross-sectional, observational studies were initiated in 2012: a physician survey and a medical records review. The first two out of three planned rounds for each study are reported.

**Methods:**

The primary objective in the physician survey was to estimate the prevalence of *KRAS* testing, and in the medical records review, it was to evaluate the effect of test results on patterns of panitumumab use. The medical records review study also included a pathologists’ survey.

**Results:**

In the physician survey, nearly all oncologists (299/301) were aware of the correct panitumumab indication and the need to test patients’ tumor *KRAS* status before treatment with panitumumab. Nearly all oncologists (283/301) had in the past 6 months of clinical practice administered panitumumab correctly to mCRC patients with wild-type *KRAS* status. In the medical records review, 97.5% of participating oncologists (77/79) conducted a *KRAS* test for all of their patients prior to prescribing panitumumab. Four patients (1.3%) did not have tumor *KRAS* mutation status tested prior to starting panitumumab treatment. Approximately one-quarter of patients (85/306) were treated with panitumumab and concurrent oxaliplatin-containing chemotherapy; of these, 83/85 had confirmed wild-type *KRAS* status prior to starting panitumumab treatment. All 56 referred laboratories that participated used a Conformité Européenne-marked or otherwise validated *KRAS* detection method, and nearly all (55/56) participated in a quality assurance scheme.

**Conclusions:**

There was a high level of knowledge amongst oncologists around panitumumab prescribing information and the need to test and confirm patients’ tumors as being wild-type *KRAS* prior to treatment with panitumumab, with or without concurrent oxaliplatin-containing therapy.

## Introduction

Anti-epidermal growth factor receptor (EGFR) monoclonal antibodies (mAbs), such as panitumumab (Vectibix^®^, a recombinant, fully human IgG2 mAb) and cetuximab (Erbitux^®^, a recombinant, chimeric mouse/human IgG1 mAb), bind with high affinity and specificity to the EGFR, and have been shown to be effective across all lines of treatment in metastatic colorectal cancer (mCRC) [1–7]. Reports of improved efficacy with panitumumab and cetuximab in patients with wild-type versus mutant or unknown *KRAS* exon 2 status [8–15] led to the requirement for physicians to determine a patient’s tumor *KRAS* mutation status prior to starting treatment with EGFR inhibitors. Physicians can now determine the most appropriate treatment option for individual patients with mCRC, based upon the molecular profile of their tumor.

Panitumumab was first approved in Europe in December 2007 as monotherapy to treat patients with wild-type *KRAS* exon 2 mCRC who had failed on prior fluoropyrimidine-, oxaliplatin-, and irinotecan-containing chemotherapy regimens, based upon phase III clinical data [6,8]. Educational materials for panitumumab have been distributed to physicians since 2009, making them aware of the appropriate prescribing information and the need for *KRAS* mutation status to be determined by an experienced laboratory prior to prescribing panitumumab. In November 2011, data from two additional phase III studies led to the European panitumumab license being expanded to include use in patients with wild-type *KRAS* exon 2 mCRC in the first-line setting combined with FOLFOX [16], and the second-line setting combined with FOLFIRI [17]. Within the panitumumab licensed indication, concurrent treatment with oxaliplatin-containing chemotherapy in patients with mutant or unknown *KRAS* mCRC status was contraindicated, due to detrimental effects on progression free survival and overall survival.

Previous physician surveys have found that in 2010, when guidelines first recommended testing for *KRAS* status [18,19], the adoption of *KRAS* testing prior to treating patients with EGFR inhibitors varied widely [20–22]. In 2010, 73% (326/448) of participating physicians in Europe reported undertaking appropriate *KRAS* testing when mCRC was diagnosed, compared with 63% (160/256) in Latin America and 20% (28/139) in Asia [20]. However, there was a rapid and widespread adoption of *KRAS* testing prior to treating patients with EGFR inhibitors (3% in 2008; 47% in 2009; 69% in 2010), with results available quickly (within 15 days) for more than 80% of patients [20]. In the US, a survey carried out in 2010 reported that of 1,242 physicians responding, only one-fifth of those who had treated mCRC had ordered or recommended *KRAS* testing [21]. In contrast in another study, a more targeted identification of oncologists treating mCRC found that in 2010 all oncologists (34/34) tested tumor *KRAS* status [22].

Medical records review studies carried out in 2010 reported that over 94% of patients were being tested for tumor *KRAS* status prior to being treated with EGFR-targeted therapies [23,24]. In 93 sites from France, 94% of 1,044 patients being treated with cetuximab for CRC had been tested for tumor *KRAS* status, with wild-type *KRAS* confirmed in 95% of tested patients [23]. In the US, there was rapid uptake of *KRAS* tumor testing by 2010 in one study, with 97% of 1,188 patients with mCRC being tested in 2010 compared with 7% of patients being tested in 2006 [24].

In order to evaluate physician awareness of the prescribing information for panitumumab and how physicians prescribe panitumumab in Europe, two studies were initiated in 2012: a physician survey evaluating oncologists’ knowledge of the licensed indication for panitumumab, and a medical records review of patients with mCRC treated with panitumumab to evaluate how oncologists prescribe panitumumab. The first two rounds focused upon *KRAS* exon 2 status and are reported here. In July 2013, based upon improved outcomes in patients with wild-type versus mutant or unknown tumor *RAS* status [25], the European license for panitumumab was refined to target panitumumab treatment to patients with wild-type *RAS* mCRC. The ongoing third and final rounds of each study are focusing on tumor *RAS* (*KRAS* exons 2/3/4 and *NRAS* exons 2/3/4) testing, and will be reported separately in a future publication. Both studies will help to understand the level of acceptance of tumor *KRAS/RAS* testing by clinicians during their decision processes around treating with panitumumab.

## Materials and Methods

### Study participants

A physician survey and a medical records review were designed in Europe: both were multi-country, cross-sectional, observational studies with practicing oncologists who had prescribed panitumumab to mCRC patients in the previous 6 months. Rounds 1 and 2 (evaluating *KRAS* testing) were conducted between September 2012 and December 2013. Round 3 (evaluating *RAS* testing) is currently being conducted for both studies in 2014 and 2015.

In Round 1, physicians in five countries (France, Germany, Italy, Spain, and the Czech Republic) were invited to participate. Four additional countries (Belgium, Denmark, The Netherlands, and Sweden) were included in Round 2 to increase representation of countries in Europe. In both rounds of each study, physicians were screened by telephone using standardized questionnaires to determine eligibility.

The sampling list used in Round 1 was based on a healthcare industry database provider (Cegedim) medical marketing database and included physician contact details that were not filtered by specialty. Physicians were randomly selected for inclusion in the study from this list. The inclusion of physicians who were not oncologists in the study population partly explains the low response rate observed in Round 1 of the study. In Round 2 of the medical chart review the sampling list used included the medical marketing database filtered by specialty in addition to lists of colorectal cancer physicians. This approach allowed for a more targeted sampling of oncologists and led to a higher physician response rate in Round 2 of the chart review than observed in Round 1.

In order to obtain data on pathology testing, participating oncologists in the medical records review study were asked permission for the investigator to approach the pathology laboratories which carried out *KRAS* mutation tests, in order to send them a pathology survey to complete.

Study protocols and informed consent forms (ICF) were reviewed and approved by the local Institutional Review Board or Ethical Review Board in each country before the studies began, as required. As agreed with the ethical committees, in France and The Netherlands signed ICFs were not required, only verbal ICF (recorded in the patient’s file). ICFs were required in Germany (reviewed and approved by the Ethik-Kommission der Bayerischen Landesärztekammer), Italy (reviewed and approved by the EC Azienda AOU S. Maria della Misericordia di Udine), Spain (reviewed and approved by the Comite Etico de Investigacion Clinica H.G.U. Gregorio Maranon), Czech Republic (reviewed and approved by the Eticka Komise Masarykova Onkologickeho Ustavu, Brno), Belgium (reviewed and approved by the Medical Ethics Committee des Cliniques Saint-Joseph, Liege) and Sweden (reviewed and approved by the Regional Ethic Committee Board in Sweden—Karolina Institutet/ Solna). Ethics Committee approval was not required in Denmark, but a signed ICF was required in all Danish sites.

### Inclusion criteria for participating physicians

#### Physician survey

Practicing oncology specialists were eligible if they had treated at least five (Round 1) or three (Round 2) new or continuing patients with mCRC in the last 3 months and prescribed panitumumab in the previous 6 months.

#### Medical records review

Practicing oncologists who had treated at least five (Round 1) or three (Round 2) new or continuing patients with mCRC in the last 3 months, and had prescribed panitumumab to treat new or continuing patients with mCRC in the past 6 months were eligible. Only one oncologist per medical center could participate for each round of the study.

### Exclusion criteria for participating physicians

In Round 2 of each study, physicians were excluded if they had already taken part in Round 1. Physicians were excluded from the physician survey if they had taken part in the medical record review (Round 1 or 2). Physicians were excluded from the medical record review if they had taken part in the physicians survey (Round 1 only).

Note that in Round 2 of the medical records review, a more targeted sampling of oncologists was introduced with physicians from specialties that do not treat mCRC being excluded. In addition, in both studies, the number of new or continuing patients with mCRC requiring treatment in the last 3 months was reduced from at least five in Round 1, to at least three in Round 2. These changes were introduced in order to improve physician response rates.

### Inclusion criteria for patients

In the medical records review, eligible patients must have received panitumumab (outside of a clinical trial setting) for the treatment of mCRC during the 6-month period prior to the time when medical records were obtained, and have provided written consent to allow access to their medical records (if local laws required it). Informed consent was obtained from a legally acceptable representative of deceased patients, where necessary, to allow access to their medical records for the purpose of this study.

### Study objectives

#### Physician survey

The primary objective is to assess oncologists’ knowledge of the appropriate licensed indication for panitumumab in mCRC patients regarding tumor *KRAS* status in selected European countries. Specific primary objective measures are to evaluate oncologists’ knowledge: of the licensed indication for panitumumab, which is for the treatment of patients with wild-type *KRAS* mCRC in first line combined with FOLFOX, in second line combined with FOLFIRI in patients who have received first–line fluoropyrimidine-based chemotherapy (excluding irinotecan), and as monotherapy after failure of fluoropyrimidine-, oxaliplatin-, and irinotecan-containing chemotherapy regimens; that testing tumor *KRAS* mutation status should be performed prior to starting treatment with panitumumab; that panitumumab is not indicated for mCRC patients with mutant or unknown *KRAS* tumor status; that panitumumab should not be administered in combination with oxaliplatin-containing chemotherapy in mCRC patients with mutant or unknown *KRAS* tumor status. An additional objective measure is to characterize the results of the survey in each round to evaluate whether there are any differences between rounds.

#### Medical records review

The primary objective is to estimate the prevalence of *KRAS* testing and the impact of *KRAS* test results on patterns of panitumumab use in patients with mCRC treated with panitumumab in selected European countries, following changes in the licensed indication regarding the risk of panitumumab use in mCRC patients with mutant *KRAS* tumors. The primary objective measures include: the estimated proportion of patients with mCRC tested for *KRAS* status prior to treatment with panitumumab; the proportion of tested patients treated with panitumumab who had mutant, wild-type, or unknown tumor *KRAS* status; the estimated proportion of patients with mCRC tested for *KRAS* status prior to treatment with panitumumab who were treated concurrently with oxaliplatin-containing chemotherapy; the proportion of tested patients treated with panitumumab concurrently with oxaliplatin-containing chemotherapy who had mutant, wild-type, or unknown tumor *KRAS* status. Secondary objective measures include the estimated proportion of: oncologists who agree to participate in the study; oncologists who conduct a *KRAS* test prior to prescribing panitumumab (in those patients with mCRC who had been treated with panitumumab); laboratories that tested mCRC *KRAS* status for those patients who were treated with panitumumab who i) participate in the European Society of Pathology (ESP) Quality Assurance (QA) Scheme, or are certified by an ESP-approved accreditation body, and ii) use a Conformité Européenne (CE)-marked or otherwise validated *KRAS* detection method. An additional objective measure is to characterize the results of each round of the medical records review, and to evaluate whether there are any differences between rounds.

### Data collection

#### Physician survey

In each round of survey, telephone interviews with eligible participating oncologists were conducted by trained study staff using a standardized questionnaire and following consistent procedures and data collection. The questionnaire was carefully translated to ensure that the content and the way of asking questions was consistent across countries. Two pilot interviews were conducted in each country in Round 1 to ensure that all questions were well targeted and correctly understood; questions were reviewed and modified as needed, depending on the outcome of the pilot interviews.

#### Medical records review

Medical information relating to panitumumab (including but not limited to 3 months before and after the first dose of panitumumab) and tumor *KRAS* testing was abstracted and anonymized at the site from medical records for transfer into standardized electronic case report forms. Abstracted and anonymized data were checked by trained data extractors prior to being included in the data analysis. Two pilot abstractions were conducted in each country prior to Round 1, to ensure all questions were well targeted and correctly understood, with modifications made as required.

### Statistical analysis

In both studies, no formal hypothesis testing was conducted and the data analysis was descriptive in nature. For the categorical study endpoints described above, the count and proportion (%) in each category, based on the appropriate denominator, were calculated. The 95% confidence intervals for the proportions are based on a normal approximation to the binomial distribution.

## Results

### Physician response rate and baseline characteristics

#### Physician survey

**Fig 1:**
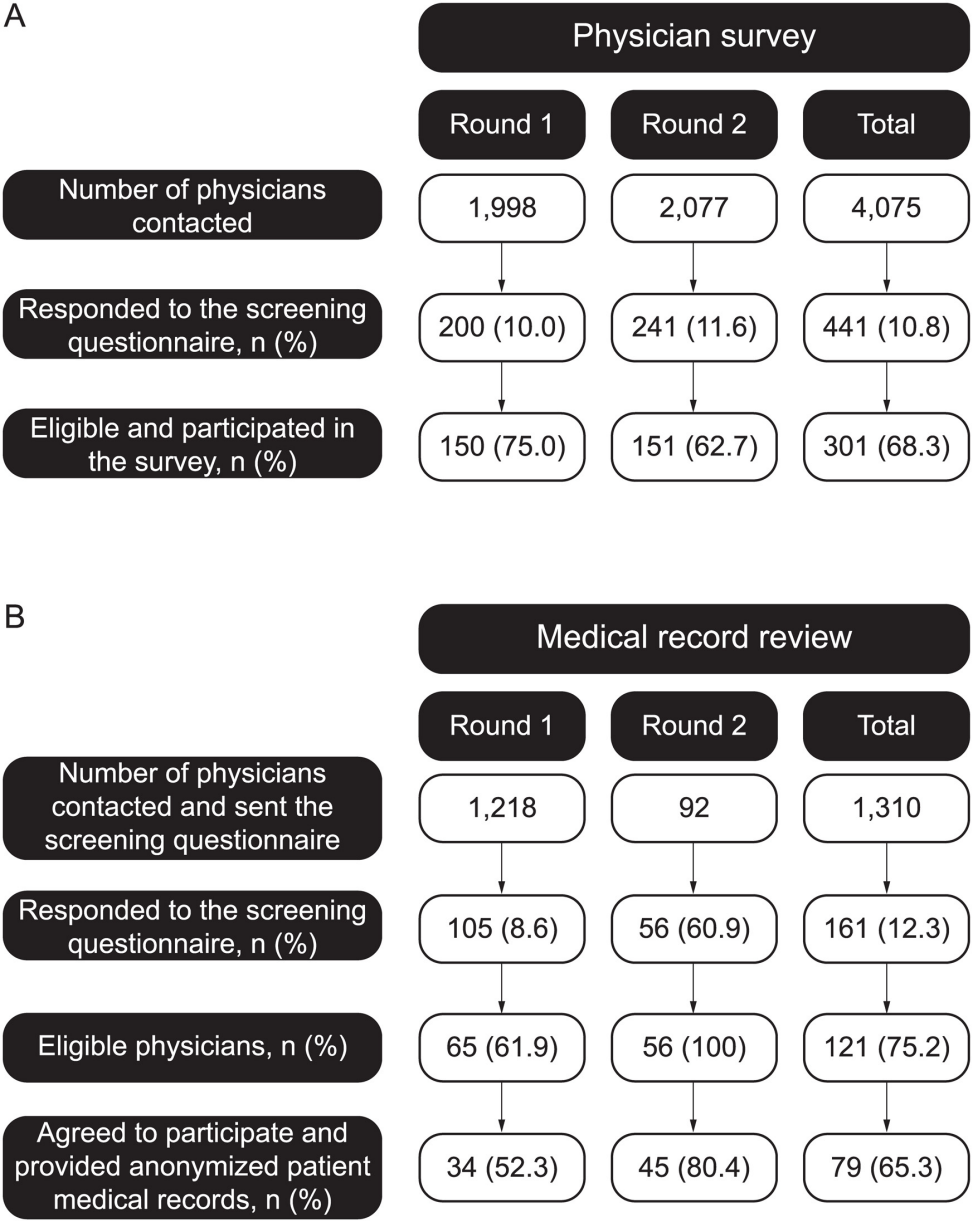
Physician disposition for the A) physician survey and B) medical records review studies.

There were low responses in the first two rounds of the physician survey, with 10.8% (441/4,075) of physicians responding to the screening questionnaire (Fig 1A).

In Rounds 1 and 2 combined, 301 eligible physicians who responded to the screening questionnaire agreed to participate (Fig 1A), and their characteristics were generally similar between rounds (Table 1). Participating oncologists were mainly from university/training hospitals (41.5%; n = 125) and general/regional hospitals (30.2%; n = 91), with the median (Q1–Q3) number of in-patient beds being 400 (100–773) (Table 1). The median (Q1–Q3) number of years of experience as a practicing oncologist specialist was 11 (7–16), with oncologists treating 40 (25–70) mCRC patients in the last 3 months (Table 1). Over three quarters (78.1%; 235/301) of participating oncologists recalled having received educational material regarding *KRAS* testing.

**Table 1:** Oncologist demographics in the physician survey and medical records review studies.

	Physician survey			Medical records review		
	Round 1	Round 2	Total	Round 1	Round 2	Total
	N = 150	N = 151	N = 301	N = 34	N = 45	N = 79
Country, n (%)						
France	50 (33.3)	39 (25.8)	89 (29.6)	14 (41.2)	9 (20.0)	23 (29.1)
Germany	35 (23.3	44 (29.1)	79 (26.2)	15 (44.1)	11 (24.4)	26 (32.9)
Italy	28 (18.7)	18 (11.9)	46 (15.3)	1 (2.9)	5 (11.1)	6 (7.6)
Spain	25 (16.7)	19 (12.6)	44 (14.6)	3 (8.8)	5 (11.1)	8 (10.1)
Czech Republic	12 (8.0)	6 (4.0)	18 (6.0)	1 (2.9)	4 (8.9)	5 (6.3)
Belgium		6 (4.0)	6 (2.0)		3 (6.7)	3 (3.8)
Denmark		5 (3.3)	5 (1.7)		3 (6.7)	3 (3.8)
Netherlands		9 (6.0)	9 (3.0)		2 (4.4)	2 (2.5)
Sweden		5 (3.3)	5 (1.7)		3 (6.7)	3 (3.8)
Type of institution, n (%)						
General or regional						
hospital	52 (34.7)	39 (25.8)	91 (30.2)	8 (23.5)	12 (26.7)	20 (25.3)
Oncology clinic/institute	16 (10.7)	14 (9.3)	30 (10.0)	0	7 (15.6)	7 (8.9)
Private clinic/hospital	27 (18.0)	23 (15.2)	50 (16.6)	20 (58.8)	13 (28.9)	33 (41.8)
University/training						
hospital	51 (34.0)	74 (49.0)	125 (41.5)	4 (11.8)	9 (20.0)	13 (16.5)
Other	4 (2.7)	1 (0.7)	5 (1.7)	2 (5.9)	4 (8.9)	6 (7.6)
Size of intitution (number of inpatient beds)						
n	141	151	292	28	42	70
Mean (SD)	383.4 (377.1)	596.5 (515.5)	493.6 (465.6)	319.3 (496.1)	315.3 (549.9)	316.9 (525.4)
Median (Q1–Q3)	300.0 (50.0–600.0)	550.0 (200.0–800.0)	400.0 (100.0–772.5)	69.5 (7.0–350.0)	50.0 (17.0–400.0)	50.0 (17.0–400.0)
Number of years' experience as a practicing oncologist						
n	149	151	300	34	45	79
Mean (SD)	12.8 (7.7)	12.2 (6.5)	12.5 (7.1)	12.3 (6.1)	18.2 (9.1)	15.7 (8.5)
Median (Q1–Q3)	12.0 (7.0–16.0)	11.0 (7.0–15.0)	11.0 (7.0–16.0)	12.5 (6.0–16.0)	19.0 (13.0–24.0)	15.0 (10.0–20.0)
Number of mCRC patients treated by the oncologist in the previous 3 months						
n	150	151	301	34	45	79
Mean (SD)	58.7 (58.3)	59.4 (57.3)	59.0 (57.7)	75.1 (96.3)	45.1 (33.1)	58.0 (69.0)
Median (Q1–Q3)	45.0 (20.0–60.0)	40.0 (25.0–70.0)	40.0 (25.0–70.0)	47.5 (20.0–90.0)	30.0 (20.0–60.0)	36.0 (20.0–70.0)
Number of mCRC patients treated with panitumumab in the last 6 months						
n	NsR	NR	NR	34	45	79
Mean (SD)				9.4 (6.0)	9.0 (10.9)	9.2 (9.1)
Median (Q1–Q3)				8.0 (6.0–10.0)	6.0 (5.0–8.0)	6.0 (5.0–10.0)

mCRC, metastatic colorectal cancer; NR, not recorded; SD, standard deviation.

#### Medical records review

In Round 1, low responses to the screening questionnaire (8.6%; 105/1,218) were observed (Fig 1B). However, in Round 2 response rates increased (60.9%; 56/92) as a result of changes in methodology and inclusion criteria outlined in the Methods section, with all 56 oncologists responding to the screening questionnaire being eligible (40 were not eligible in Round 1).

Seventy-nine eligible physicians who responded to the screening questionnaire agreed to participate (Fig 1B). The characteristics of those who participated were generally similar in Rounds 1 and 2, though the proportion based in private practice halved from 58.8% (20/34) in Round 1 to 28.9% (13/45) in Round 2 (Table 1). The median (Q1–Q3) number of years of experience as a practicing oncologist specialist was 13 (6–16) and 19 (13–24) years in Rounds 1 and 2, respectively.

Three hundred and six patient records from the 79 participating oncologists in both rounds of the medical records review were evaluated. Two-thirds of patients were male (66.7%; 204/306), and approximately one-quarter (23.9%; 73/306) were 75 years of age or older (Table 2). Just over one-quarter were receiving concurrent panitumumab and oxaliplatin-containing therapy (27.8%; 85/306) (Table 2).

**Table 2:** Patient demographics in the medical records review study.

**All patients**	**Round 1**	**Round 2**	**Total**
	**N = 155**	**N = 151**	**N = 306**
Sex—male, n (%)	96 (61.9)	108 (71.5)	204 (66.7)
Age (years)—mean (SD)	66.1 (11.0)	66.8 (10.9)	66.4 (10.9)
Age ≥65 years, n (%)	93 (60.0)	96 (63.6)	189 (61.8)
Age ≥75 years, n (%)	39 (25.2)	34 (22.5)	73 (23.9)
**Patients receiving concurrent oxaliplatin** ^a^	**Round 1**	**Round 2**	**Total**
	**N = 53**	**N = 53**	**N = 85**
Sex—male, n (%)	39 (73.6)	26 (81.3)	65 (76.5)
Age (years)—mean (SD)	63.5 (9.5)	64.4 (13.7)	63.8 (11.2)
Age ≥65 years, n (%)	29 (54.7)	19 (59.4)	48 (56.5)
Age ≥75 years, n (%)	7 (13.2)	8 (25.0)	15 (17.6)

SD, standard deviation.

^a^Received oxaliplatin-containing chemotherapy during the interval from 7 days prior to the date of first dose of panitumumab, until 7 days after the last dose of panitumumab.

### Physician’s understanding of who, and how, to treat with panitumumab

#### Physician survey results: Awareness of the licensed indication and testing for KRAS status before starting treatment with panitumumab

Nearly all oncologists were aware of the need to test patients with mCRC for *KRAS* status before treatment with panitumumab (99.3%; 299/301) (Table 3). Of the two remaining oncologists, one did not consider *KRAS* testing to be appropriate, and the other provided no response. Nearly all oncologists (99.0%; 298/301) were aware of the correct indication for panitumumab for treatment of mCRC patients with wild-type *KRAS* tumors; two oncologists responded for the treatment of patients with mutant mCRC *KRAS* tumors, and one gave an invalid multiple response. Almost all (97.7%; 294/301) oncologists were aware of all their patients’ tumor *KRAS* status prior to treatment with panitumumab (Table 3). Six oncologists were unaware of their patients’ tumor *KRAS* mutation status prior to treatment with panitumumab, and the other gave a missing response.

**Table 3:** Outcomes of testing for *KRAS* exon 2 in the physician survey study.

	n (%)		
	(95% CI: %)		
	**Round 1**	**Round 2**	**Total**
**All oncologists**	**N = 150**	**N = 151**	**N = 301**
Aware *KRAS* testing should be performed prior to	149 (99.3)	150 (99.3)	299 (99.3)
initiation of panitumumab^a^	(98.0–100.0)	(98.0–100.0)	(98.4–100.0)
Aware of the correct indication for panitumumab	148 (98.7)	150 (99.3)	298 (99.0)
for treatment of mCRC patients with wild-type	(96.8–100.0)	(98.0–100.0)	(97.9–100.0)
*KRAS* tumors^b^			
Aware of patients tumor *KRAS* status prior to	144 (96.0)	150 (99.3)	294 (97.7)
initiation of panitumumab treatment in the past	(92.9–99.1)	(98.0–100.0)	(96.0–99.4)
6 months of routine clinical practice^c^			
Administered panitumumab to only mCRC	143 (95.3)	140 (92.7)	283 (94.0)
patients with wild-type *KRAS* in the past 6 months	(92.0–98.7)	(88.6–96.9)	(91.3–96.7)
of routine clinical practice^d^			
**Subset of oncologists who administered**			
**panitumumab concurently with oxaliplatin-**	**Round 1**	**Round 2**	**Total**
**containing chemotherapy**	**N = 74**	**N = 90**	**N = 164**
Administered panitumumab with concurrent	70 (94.6)	83 (92.2)	153 (93.3)
oxaliplatin-containing chemotherapy to only	(89.4–99.7)	(86.7–97.8)	(89.5–97.1)
mCRC patients with wild-type *KRAS* in the past			
6 months of routine clinical practice^e^			

CI, confidence interval; mCRC, metastatic colorectal cancer.

^a^One oncologist responded that *KRAS* testing was not appropriate (Round 1), and one oncologist did not respond (Round 2).

^b^Two oncologists responded for treatment of patients with mutant mCRC *KRAS* tumors (one in each round), and one gave an invalid multiple response (Round 1).

^c^Six oncologists were unaware of patients’ tumor *KRAS* status prior to initiation of panitumumab treatment (Round 1), and one oncologist gave a missing response (Round 2).

^d^Fifteen oncologists had administered panitumumab to mCRC patients with mutant *KRAS* tumors or with tumor *KRAS* status unknown (seven in Round 1 and eight in Round 2), two oncologists gave a not sure response (Round 2), and one oncologist did not give a response (Round 2).

^e^Ten oncologists had administered panitumumab with concurrent oxaliplatin-containing chemotherapy to mCRC patients with mutant *KRAS* tumors or with tumor *KRAS* status unknown (three in Round 1 and seven in Round 2), and one oncologist gave a not sure response (Round 1).

*Correct administration of panitumumab*: Nearly all oncologists (94.0%; 283/301) had administered panitumumab correctly to all their mCRC patients in the past 6 months of clinical practice (those with known wild-type *KRAS* status prior to first dose of panitumumab) (Table 3). Of the responses from the remaining 18 oncologists, 15 (5.0%) had administered panitumumab to at least one patient with mutant or unknown *KRAS* status in the past 6 months of clinical practice, two (0.7%) were unsure, and one (0.3%) gave a missing response. The most frequent influencing factors cited for treating patients with mutant *KRAS* status were the patient’s medical status (n = 8) and other (n = 6: not concerned with *KRAS* mutation type [n = 3]; administered when *KRAS* status was unknown [n = 1]; special [13] mutation [n = 1]; good results with other patients [n = 1]). The most frequent influencing factors cited for treating patients with *KRAS* status unknown were time to obtain *KRAS* results (n = 6), tissue not available / insufficient tissue available (n = 4) and patient’s status or medical condition (n = 4). It should be noted that multiple reasons could be given.

Of the 301 participating oncologists, 164 (54.5%) had administered panitumumab concurrently with oxaliplatin-containing therapy to at least one mCRC patient in the past 6 months of routine clinical practice (Table 3). Of these, 10 (6.1%) oncologists had administered panitumumab simultaneously with oxaliplatin-containing chemotherapy to mCRC patients with *KRAS* status mutant or unknown, and one oncologist was unsure (Table 3). There was no indication from the free-text responses that these oncologists were unaware of the contraindication of administering panitumumab simultaneously with oxaliplatin-containing chemotherapy to mCRC patients with tumor *KRAS* status unknown.

*Differences in survey responses between rounds*: There were no noticeable differences in the results obtained for the physician survey between Rounds 1 and 2.

### How oncologists treat patients with panitumumab based upon *KRAS* testing

#### Medical records review results: Proportion of oncologists testing patients’ tumors for KRAS status prior to treatment with panitumumab

Nearly all oncologists (97.5%; 77/79) conducted a *KRAS* mutation test for all their mCRC patients prior to prescribing panitumumab.

Almost all patients (98.7%; 302/306) had been tested for *KRAS* mutation status prior to their first panitumumab dose, with wild-type *KRAS* mutation status confirmed in 97.7% (299/306) of patients (Table 4). Four patients (1.3%; all from two oncologists) had not been tested for *KRAS* mutation status prior to their first dose of panitumumab. One patient had a *KRAS* test result (wild-type) dated after the first panitumumab dose (this patient was also receiving concurrent oxaliplatin-containing chemotherapy). Three patients had no *KRAS* test result available (tissue was not available/insufficient tissue available [1 patient], unaware of the need for *KRAS* test [1 patient]; patient’s situation [1 patient]).

**Table 4:** Outcomes of testing for *KRAS* exon 2 in the medical records review study.

	n (%)		
	(95% CI: %)		
	**Round 1**	**Round 2**	**Total**
**All patients**	**N = 155**	**N = 151**	**N = 306**
Tested for *KRAS* status prior to first dose of	153 (98.7)	149 (98.7)	302 (98.7)
panitumumab^a^	(96.9–100.0)	(96.9–100.0)	(97.4–100.0)
Wild-type *KRAS* test result confirmed prior to	152 (98.1)	147 (97.4)	299 (97.7)
first dose of panitumumab^b^	(95.9–100.0)	(94.8–99.9)	(96.0–99.4)
**Subset of patients treated with concurrent**	**Round 1**	**Round 2**	**Total**
**oxaliplatin-containing chemotherapy** ^c^	**N = 53**	**N = 32**	**N = 85**
Tested for *KRAS* status prior to first dose of	51 (96.2)	32 (100)	83 (97.6)
panitumumab^d^	(91.1–100.0)	100.0–100.0)	(94.4–100.0)
Wild-type *KRAS* test result confirmed prior to	51 (96.2)	32 (100)	83 (97.6)
first dose of panitumumab	(91.1–100.0)	(100.0–100.0)	(94.4–100.0)

CI, confidence interval; mCRC, metastatic colorectal cancer.

^a^Three patients did not have a *KRAS* test performed (one in Round 1 and two in Round 2), and one patient had a *KRAS* test performed after the first dose of panitumumab (Round 1).

^b^Two patients had a mutant *KRAS* test result confirmed prior to first dose of panitumumab (one in Round 1 and one in Round 2) and one patient had an unknown *KRAS* test result (Round 2).

^c^Received oxaliplatin-containing chemotherapy at any time during the interval from 7 days prior to the date of first dose of panitumumab, until 7 days after the last dose of panitumumab.

^d^One patient did not have a *KRAS* test performed (Round 1), and one patient had a *KRAS* test performed after the first dose of panitumumab (Round 1).

Three (1.0%) of the 302 patients tested for *KRAS* tumor status prior to their first dose of panitumumab had mutant (n = 2) or unknown (n = 1) *KRAS* status (none were receiving concurrent oxaliplatin-containing therapy (Table 4).

*Testing patients’ tumors for KRAS status prior to treatment with panitumumab and concurrent oxaliplatin-containing chemotherapy*: Approximately one quarter of patients (27.8%; 85/306) were treated with panitumumab and concurrent oxaliplatin-containing chemotherapy, with nearly all of these patients (97.6%; 83/85) having a confirmed wild-type *KRAS* result prior to starting treatment (Table 4).

*Laboratory use of validated KRAS detection methods*: Participating oncologists were asked to name the corresponding laboratories that analyzed the tumor samples. Of the 95 named laboratories, 56 (58.9%) responded to the survey. All 56 laboratories (100%) used a CE-marked or otherwise validated *KRAS* detection method, with 34 (60.7%) laboratories using non-commercial tests that were not CE-marked, but were otherwise validated. Nearly all (98.2%; 55/56) participated in at least one QA scheme (e.g. ESP QA scheme [10 (17.9%)], Directory of Molecular Genetic External QA schemes in Europe [8 (14.3%)], United Kingdom National External QA Service [4 (7.1%)], local or regional QA schemes such as the French Gen&Tiss scheme [25 (44.6%)], or other QA schemes [21 (37.5%)]).

*Differences in medical record review responses between rounds*: There were no noticeable differences in the results obtained for the medical records review study between Rounds 1 and 2.

## Discussion

In the physician survey, amongst participating oncologists, nearly all those surveyed in 2012 and 2013 (99.3%; 299/301) were aware of the need to test tumor *KRAS* status before administration of panitumumab to patients with mCRC. Nearly all oncologists (94.0%; 283/301) had administered panitumumab correctly to all of their mCRC patients with wild-type *KRAS* status, according to the label, in the past 6 months of clinical practice. In the medical records review, nearly all oncologists (97.5%) conducted a *KRAS* test for all of their patients prior to prescribing panitumumab. Importantly, tumor *KRAS* status was known in nearly all (98.7%) patients prior to prescribing panitumumab (with or without oxaliplatin-containing therapy), with tumor wild-type *KRAS* confirmed in 97.7% of patients. All corresponding laboratories that responded to the pathologist survey used validated *KRAS* testing methods, and regularly checked their testing methods versus QA schemes in place.

Fifteen of the 301 oncologists in the physician survey said that in the last 6 months of clinical practice they had administered at least once panitumumab to mCRC patients when the *KRAS* result was mutant or unknown. This proportion (5%) is higher in the survey than in the chart review study in which 1% of the patients had mutant or unknown *KRAS* results at the initiation of panitumumab treatment. The physician survey records the number of physicians who had administered panitumumab outside the label indication once or more in the last 6 months and therefore, did not capture the number patients treated according to the label as the chart review did. Oncologists quoted a number of reasons for prescribing panitumumab (with or without concurrent oxaliplatin-containing therapy) to patients with mutant or unknown *KRAS* status, with the most frequent influencing factors being the patients’ medical condition, that they were unconcerned by *KRAS* mutation status, the availability of tumor tissue, and the time taken for *KRAS* test results to be received. It appears that on rare occasions physicians are using their clinical judgment and they do not adhere with the guidelines despite that they seem to have a good knowledge of the label indication. Characteristics of patients such as co-morbidity is a factor that has been shown in the past to influence physicians implementation of clinical guidelines [25].

Low response rates are not uncommon for physician telephone surveys. A physician survey carried out in 12 countries assessing their knowledge and application of COPD showed that contacting physicians by telephone had low response rate (USA 10%, Italy 21%) [26]. A telephone survey amongst neurologists in 12 European countries to assess awareness of clinical guidelines had a response rate of 27.2% [27]. Low response rates also occur in chart review studies, with 10% of invited physicians participating in a US-based community chart review study [28], and 34% of invited urologists participating in a retrospective on-line chart review [29].

The sampling list used for the physicians’ survey and Round 1 of chart review was based on a medical marketing database and included physician contact details that were not filtered by specialty. The inclusion of physicians who were not oncologists in the initial study population may give an underestimate of the true response rate observed in the study. For Round 2 of the chart review study a more targeted method of sampling was used where the medical marketing database was filtered by specialty. With the different sampling methods used and the similarities in baseline characteristics of physicians between rounds, the results are considered to be representative of oncologist prescribing practice in Europe and any perceived selection bias resulting from the low response rate is unlikely to be impacting the study results.

Responding and non-responding physicians have been found to have similar demographic characteristics, perhaps due to the high level of homogeneity in the physician population (comparable education, socio-economic status, etc.) [30]. In the physician survey, more participating oncologists had ≥10 years’ experience (62.1% [187/301] vs 42.7% [44/103], respectively) and had treated ≥40 mCRC patients in the previous quarter (58.1% [175/301] vs 12.6% [13/103], respectively) than screened oncologists who were not eligible for the study. Approximately three quarters of physicians could recall receiving educational materials on *KRAS* testing and this result was encouraging. Overall, we consider that the participating oncologists were likely to be representative of those treating mCRC patients in the EU.

Within the EU, individual countries are likely to have their own strategies for improving the care of patients with mCRC. In general there should be equal access to treatments and a need to develop platforms that enable patients to receive the most appropriate treatment (e.g. molecular genetics and testing). Within individual countries, the proportion of patients treated in each type of facility (institution / hospital / private practice) will differ, and the use of *KRAS* testing may also vary between facility types. In the physician survey, we have shown that by 2012, the percentage of participating physicians adopting tumor *KRAS* testing was nearly 100% in Europe. Previous physician surveys in 2010 following published guidance recommending the adoption of *KRAS* testing prior to EGFR-targeted treatment [18,19] found that the range of adoption varied widely (20–100%) across countries [20–22]. In Europe in 2010, physicians used clinical judgment when deciding whether or not to test mCRC *KRAS* status prior to treatment with EGFR-targeted therapies, with 73% of participating physicians testing mCRC *KRAS* status prior to prescribing EGFR-targeted therapies [20]. Of those specialists who did not test *KRAS* status, reasons cited were that the tests were not considered relevant for the patients (47%) or the specialist was unfamiliar with the test (40%) [21]. In Round 3 of the physician survey, more information regarding influencing factors will be collected to gain as much insight as possible into why, on rare occasions, physicians might prescribe panitumumab to patients with mutant or unknown *RAS* tumor status.

The data we report in our medical records review are supported by previous studies, which showed that, in 2010, physicians were beginning to adopt *KRAS* testing prior to EGFR-targeted treatment following published guidance [23,24]. Of those specialists in France who did not test *KRAS* status, reasons for not testing were that the patient had received prior anti-EGFR-treatment (63% of patients) or there was an absence of tumor/technical issues (27% of patients) [23]. Interestingly, in the US in 2010, of those patients with mutant *KRAS* tumor status, only 86% were not treated with EGFR-targeted therapies [24], suggesting that 14% received anti-EGFR-treatment. In comparison, by 2012 in our medical records review, 1% (3/302) of patients tested for *KRAS* tumor status prior to their first dose of panitumumab had mutant or unknown *KRAS* status (none were receiving concurrent oxaliplatin-containing therapy).

We have shown that by 2012 in Europe, there was a high level of knowledge amongst oncologists around the need to test and confirm patients’ mCRC tumors as being wild-type for *KRAS* prior to treatment with panitumumab, with or without concurrent oxaliplatin-containing therapy. Nearly all oncologists surveyed followed the licensed indication for panitumumab, but a minority still used their clinical judgment when deciding whether to treat patients with mutant or unknown *KRAS* mCRC status. While their decisions conflict with current prescribing information, discussions between individual physicians and their patients around the risks and benefits (e.g. additional biopsy, denial of treatment) may contribute to these decisions. Both studies report very good compliance with the licensed indication for panitumumab in terms of *KRAS* testing at the time of Rounds 1 and 2 of the studies. There was also a very good level of knowledge amongst oncologists around the need to test and confirm wild-type *KRAS* tumor status prior to treatment with panitumumab, with or without concurrent oxaliplatin-containing therapy. Nearly all patients treated with panitumumab (with or without concurrent oxaliplatin-containing therapy) had wild-type *KRAS* tumor status confirmed prior to first dose. It is important that tests for predictive molecular markers such as *KRAS* are carried out using validated tests in accredited laboratories that have quality controls in place. All participating laboratories used a CE-marked or otherwise validated *KRAS* detection method, and nearly all participated in a QA scheme.

In Round 3 of the physician survey and medical records review, which is evaluating knowledge of the need to test for an expanded number of *RAS* mutations prior to treating with panitumumab, the physician questionnaires will capture additional free-text details to describe the oncologists’ rationale that led to patients with mutant or unknown tumor *RAS* status receiving panitumumab. Distribution of the educational materials continues. Data from Round 3 for both studies evaluating *RAS* testing are awaited with interest.
